# Assessment of Situation Awareness Conflict Risk between Human and AI in Process System Operation

**DOI:** 10.1021/acs.iecr.2c04310

**Published:** 2023-02-21

**Authors:** He Wen, Md. Tanjin Amin, Faisal Khan, Salim Ahmed, Syed Imtiaz, Efstratios Pistikopoulos

**Affiliations:** † Centre for Risk, Integrity and Safety Engineering (C-RISE), Faculty of Engineering & Applied ScienceMemorial University, St. John’s, NL A1B 3X5, Canada; ‡ Mary Kay O’Connor Process Safety Center (MKOPSC), Artie McFerrin Department of Chemical Engineering, Texas A&M University, College Station, Texas 77843, United States; § Texas A&M Energy Institute, Artie McFerrin Department of Chemical Engineering, Texas A&M University, College Station, Texas 77843, United States

## Abstract

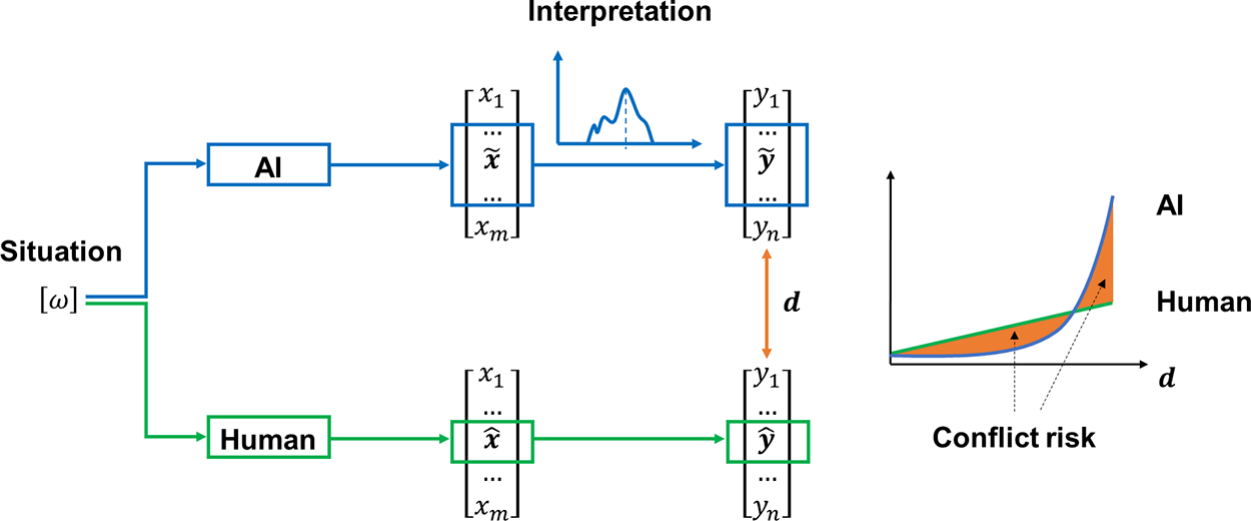

The conflict between human and artificial intelligence is a critical issue, which has recently been introduced in Process System Engineering, capturing the observation and action conflicts. Interpretation conflict is another source of potential conflict that can cause serious concern for process safety as it is often perceived as confusion, surprise, or a mistake. It is intangible and associated with situation awareness. However, interpretation conflict has not been studied with the required emphasis. The current work proposes a novel methodology to quantify interpretation conflict probability and risk. The methodology is demonstrated, tested, and validated on a two-phase separator. The results show that interpretation conflict is usually hidden, mixed, or covered by traditional faults, and noises in observation and interpretation, including sensor faults, logic errors, cyberattacks, human mistakes, and misunderstandings, may easily trigger interpretation conflict. The proposed methodology will serve as a mechanism to develop strategies to manage interpretation conflict.

## 1 Introduction

The inception of Industry 4.0 and the enhanced use of digital technologies and digitalization are reshaping the operation and structure of process systems.^1,2^ Effective utilization of artificial intelligence (AI) and machine learning (ML) algorithms is pivotal to ensure the successful adoption of Industry 4.0 and digitalization, and day by day, these technologies are increasingly being used in process industries.^3^ These have significantly improved the performance of sensors and controllers, model prediction accuracy, parameter estimation, and process optimization.^4^ Although process performance has notably improved due to the use of AI and ML, process safety incidents are still occurring.^5^ Excessive dependence on AI-based automated technologies may result in accidents (e.g., the 2005 Buncefield fire in the UK).^6^ However, research on industrial automation and AI is still a key area intending to assist humans in decision-making.^7,8^

Despite a growing focus on industrial automatization, human beings are yet in the loop for ensuring safety, especially in petrochemical industries.^9^ The role of AI, in most cases, is to help operators to have a better prediction of the situation. For instance, operators rely on ML algorithms to narrow down the search window in root cause diagnosis to restore the process to normal operating mode due to an abnormal operating condition.^10,11^ Therefore, deeply studying the interaction and collaboration in process systems is necessary. More specifically, it is of utmost importance to understand how AI decides and predicts the present and future by judging its surroundings and using the in-built logic.

Situation awareness (SA) – an appropriate awareness of the situation – plays a crucial role in the performance of humans and AI agents.^12^ For instance, the SA concept is widely used for aircraft safety in the aviation industry, and ill-judging a situation is one of the major reasons for aviation accidents.^13^ Although the definition of SA varies from the different scholars’ perspectives,^14^ SA is often categorized in the domain of human factor and considered a performance-related psychological concept. A widely accepted proposition is Endsley’s three-level model: perception (level 1), comprehension (level 2), and projection (level 3).^15^ Further studies also emphasize descriptions of individual performance at an abstract and general level,^16^ with rare quantification.^17^ The extensive research is team SA models discussing information exchange and team cooperation.^18^ However, the focus of research has always been on humans, and the SA of automated systems has rarely been studied. Recent research starts to consider distributed SA,^19^ which means the entire system-level comprehension or compatible awareness in the human-intelligent distributed system. But none of these discuss the SA differences between humans and AI agents.

In Process System Engineering (PSE), the issue of SA in major chemical accidents has also received attention, and multiple case studies have been conducted.^20^ The chemical industries emphasize team SA among operators and engineers since its role in preventing catastrophic accidents is paramount.^21^ It also stresses the holistic SA and distributed SA at the system level.^22^ There has also been some progress in quantifying the impact of SA, for example, the combination of SA and the Bayesian network.^23^ Digital technology has also expanded the research in the field of SA, and some scholars have proposed to use eye tracking technology to evaluate SA.^24^ However, no notable study exists on the SA difference quantification between humans and AI in the chemical industries.

**Figure 1:**
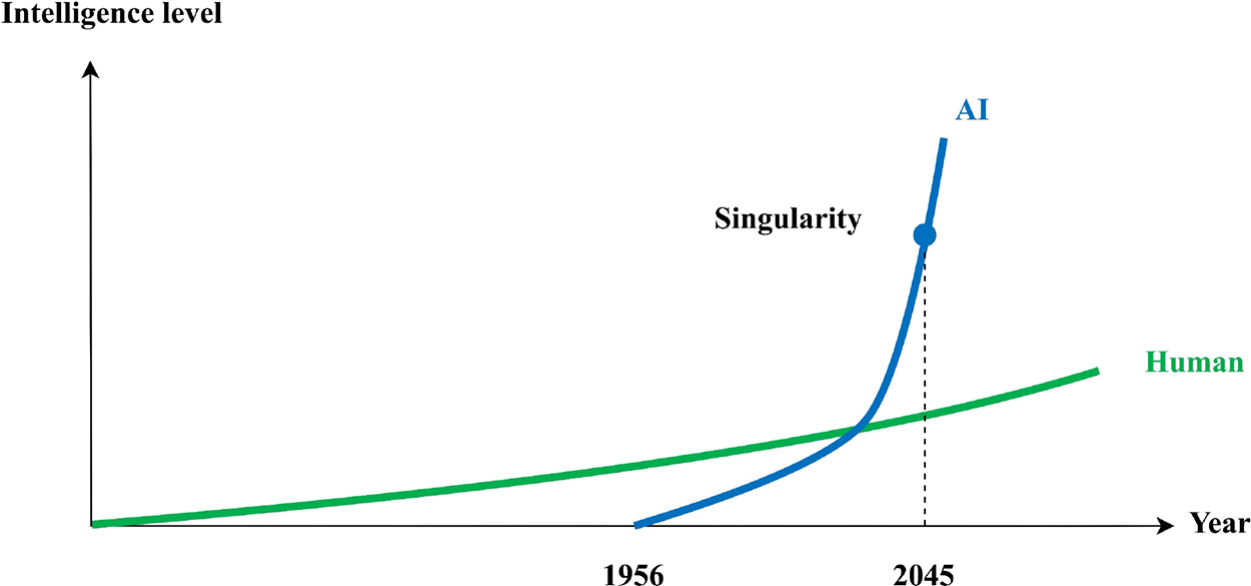
Intelligence growth of human and AI.^26^

Generally, humans are better in the context of SA due to their ability to recognize new situations earlier than AI. For instance, the recently discovered adversarial attacks showed that adding a small noise could make the AI misclassify the image.^25^ Numeric process data can also be contaminated by adding a small noise, which may mislead the logic solver or ML algorithms. Similar attacks would be easier in the form of false data injection (FDI) or denial of service (DoS). The current authors believe this performance degradation is due to AI’s loss of SA. It is alarming from a safety perspective since a controversial but widely discussed prediction is that AI will surpass all humans’ intelligence by 2045 when technology singularity occurs (Figure 1).^26,27^ The studies suggest that AI is growing exponentially, while human intelligence is growing slowly and approximately linearly. Due to this exponential growth in AI’s intelligence, it is expected to exercise more dependence on AI-based automated systems in process industries. It may be a boon because of their possible improvement in decision-making. However, it also paves the way for experiencing catastrophic accidents due to overreliance on technologies that are poor in the context of SA.

Interpretation of a situation is an important part of SA. It leads to the decision and actions suggested by the automated systems. For instance, sensors gather information about their surroundings (or situation) and make the in-built logic to interpret the situation and subsequently take decisions and actions. Similarly, human beings have a mechanism to interpret a situation and make a decision. These two decision-makers may or may not coincide in terms of their interpretation, which may result in an interpretation conflict. It is worth noting that conflict analysis in process systems between humans and machines is a novel concept that has recently been proposed.^28^ However, the authors have shown how to identify and assess risk due to observation and action conflicts without going into a detailed analysis of the interpretation conflict.

Associated with SA, interpretation conflict is also from the cognition perspective and is usually intangible. It is more likely to occur in cases of logic errors, human misunderstandings, and cyberattacks. Even when the object is imperfect or mixed with noises, an observation conflict may occur that can trigger an interpretation conflict. In the aviation industry, some situations of interpretation conflict are mode confusion^29,30^ and automation surprise.^31,32^ Also, in the context of self-driving cars, unexpected braking or changing lane confuses the driver,^33^ and the driver may have situational reactions under stress; this is an example of interpretation conflict.

Currently, the interpretation conflict between humans and AI has not been properly addressed. Though mode confusion and automation surprise have some compelling works, these studies start from the macro level, focusing on the action, not the recognition process. To the authors’ best knowledge, no work on PSE has focused on demystifying interpretation conflict between humans and AI from a safety perspective (e.g., shutting down the operation to avoid acknowledging the significant risk due to an interpretation conflict). To eliminate this gap, this study attempts to answer the following research questions: (i) what is interpretation conflict? (ii) how to identify interpretation conflict? (iii) how does interpretation conflict occur? (iv) how to assess the risk due to an interpretation conflict?

In addition, this study proposes a methodology to reveal the interpretation conflict and applies it in a two-phase separator. The novelties of this paper are the following: (i) introducing the concept of interpretation conflict, (ii) deconstructing the evolution process of interpretation conflict, (iii) exploring the impact of various noises on interpretation conflict, and (iv) developing a novel methodology to assess the risk as a result of an interpretation conflict.

The paper is arranged as follows: Section 2 describes the interpretation conflict and its evolution. Section 3 presents the proposed novel methodology to assess interpretation conflict risk. The simulation and application are described in Section 4. The results are discussed in Section 5. Finally, conclusions and future directions are shown in Section 6.

## 2 Situation Awareness Conflict (Interpretation Conflict) Evolution

### 2.1 Definition

**Figure 2:**
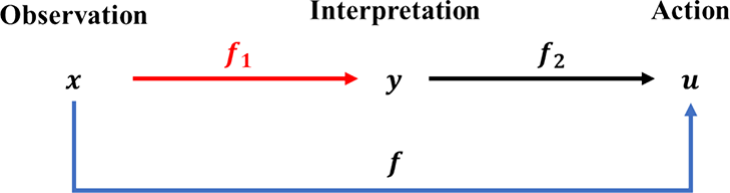
Recognition process of AI.

Conflict has been defined as the difference in the observation, interpretation, or action of one or more variables by different participants.^28^ Therefore, interpretation conflict is the difference in interpretation by different participants. Figure 2 shows the recognition process of AI to imitate human recognition.

where *x* is observation, *y* is interpretation, *u* is action, *f* is the function from observation to action, *f*_1_ is the function from observation to interpretation, and *f*_2_ is the function from interpretation to action.

The traditional control theory solves *f* with the state space equation. *f* can be destructed into two subfunctions, *f*_1_ and *f*_2_. Action is usually one-on-one with interpretation results; hence, in this study, the research focus is *f*_1_, which is situation awareness. Therefore, the fundamental cause of interpretation conflict is the difference of *f*_1_ between humans and AI.

**Figure 3:**
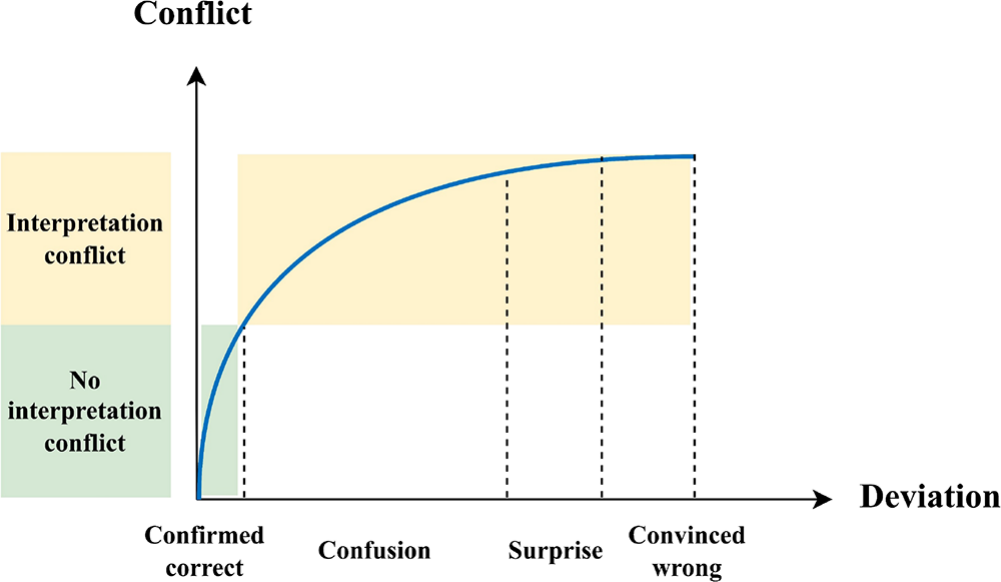
Relationship between human feeling and interpretation conflict.

As mentioned earlier, humans have a better understanding of newer situations compared to AI. Except for confirmed correct and convinced wrong, there are some gray areas of human feeling, which is the deviation between human interpretation and AI interpretation, for example, mode confusion or automation surprise, and similar feelings include hesitation, doubt, and unsureness. The relationship between such deviations and interpretation conflict is shown in Figure 3. When it is confirmed that AI is making an accurate interpretation, there should be no interpretation conflict. Otherwise, any confusion, surprise, and convinced wrong can be categorized as interpretation conflict. It is worthwhile to mention that current work is assessing interpretation conflict from a human perspective since humans have a better SA at the current level of intelligence.

### 2.2 Evolution Process and Mathematical Formulation

Although the emergence of interpretation conflict may be instant, it still has a deconstructable evolution process. First, the conflict variables are defined as follows: (i) variable of observation difference (VOD) is the difference in observation of process value from different observers; (ii) variable of interpretation difference (VID) is the difference in interpretation of process value from different interpreters; (iii) variable of action difference (VAD) is the difference in control action by different participants.^28^

**Figure 4:**
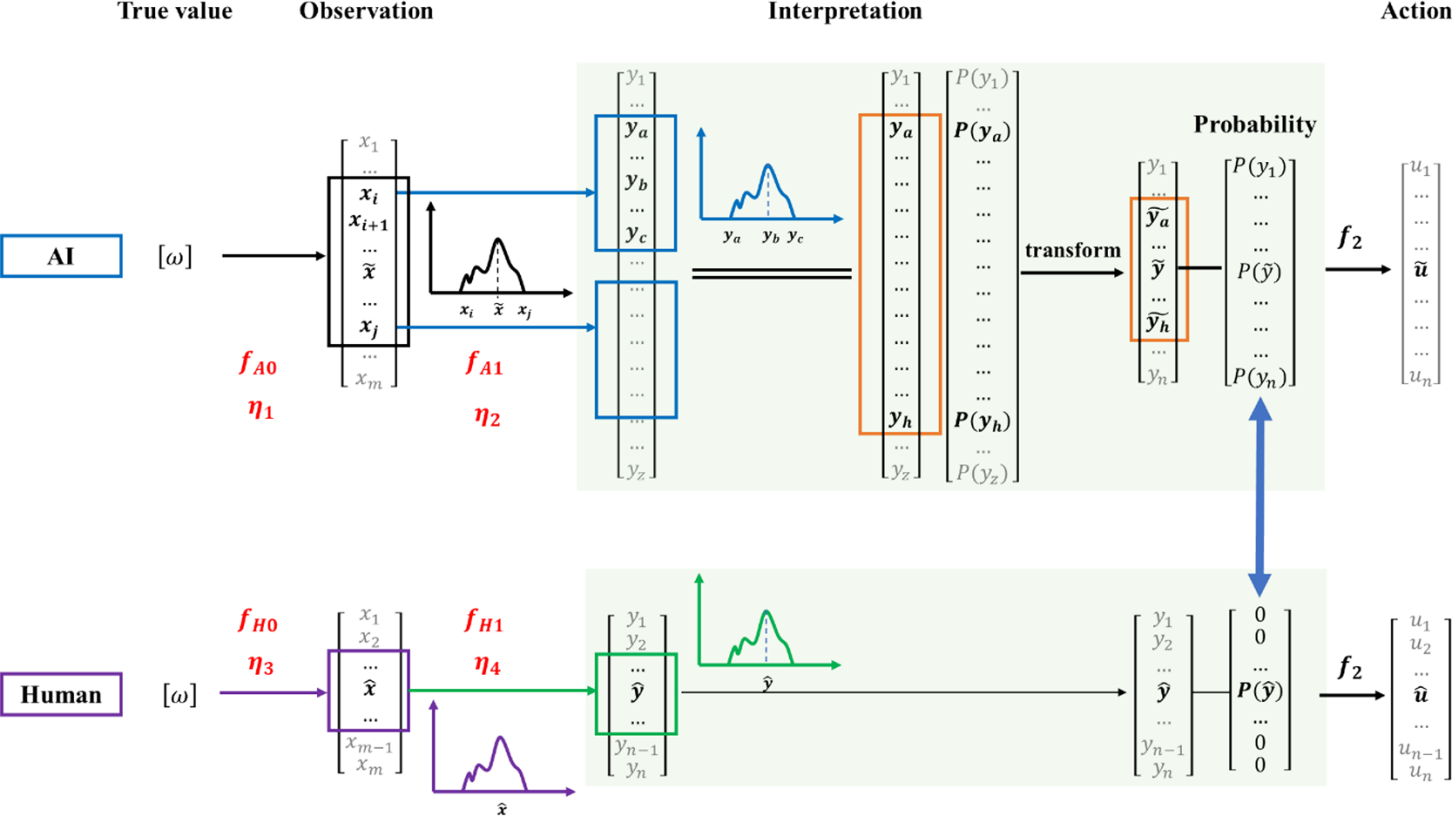
Interpretation conflict between AI and human.

In a perfect situation without noise, there is no observation conflict, no interpretation conflict, and no action conflict, which means VOD = 0, VID = 0, and VAD = 0. As different noises work on AI and humans, in most cases, there should be differences, and these are the basic causes of a conflict. Figure 4 describes how human–AI interpretation conflict occurs.

where the subscript A stands for AI and the subscript H stands for human; ω is a supposed true value; *f*_0_ is the function from true value to observation, *f*_*A*0_ is the function from true value to sensor observation, and *f*_*H*0_ is the function from true value to human observation; *f*_*A*1_ is the function from observation to interpretation of AI and *f*_*H*1_ is the function from observation to interpretation of human; η denotes noise, η_1_ is the noise in sensor observation, η_2_ is the noise in AI interpretation, η_3_ is the noise in human observation, and η_4_ is the noise in human interpretation; *m* is the observation vector size, *n* is the action vector size and the transformed interpretation vector size, *z* is the full size of the extended interpretation vector, and the other lowercase letters from *a* to *z* represent the subscripts of interpretation or observation; *x̃* is the most possible sensor observation, *ỹ* is the most possible AI interpretation, and *ũ* is the most possible AI action; *x̂* is the most possible human observation, *ŷ* is the most possible human interpretation, and *û* is the most possible human action.

For AI, given a true value, ω, as there are noises, η_1_ in observation to affect *f*_*A*0_, the noises can be measurement error by sensor, sensor fault, or FDI on sensor; also, there are noises, η_2_ in interpretation, which affects *f*_*A*1_, and the noises can be logic error, adversarial attack, FDI on controller, or DoS. Therefore, the observation, interpretation, and action equations of AI will be
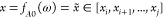
1
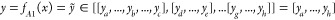
2
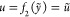
3

As one observation may correspond to multiple possible interpretations, the interpretation vector becomes longer; then, it needs to be transformed to the same size as the action vector. The range  will be transferred to 

For humans, usually, SA is an instant and straightforward process. Humans may have an estimated range of observations and then give the most possible guess; similarly, humans will give several corresponding interpretations and then make a clear choice directly, though the whole process is unknown. As there is noise, η_3_ in observation, and the noise is mostly human mistake or measurement error (by equipment or eyes), there is noise, η_4_ in interpretation, and the noise is mostly human misunderstanding. Therefore, the observation, interpretation, and action equations of human will be
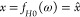
4
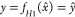
5
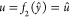
6

Consequently, VOD, VID, and VAD can be represented by eqs 7–9, respectively.
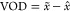
7
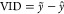
8
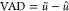
9

## 3 Proposed Methodology to Assess Interpretation Conflict Risk

### 3.1 General Description

**Figure 5:**
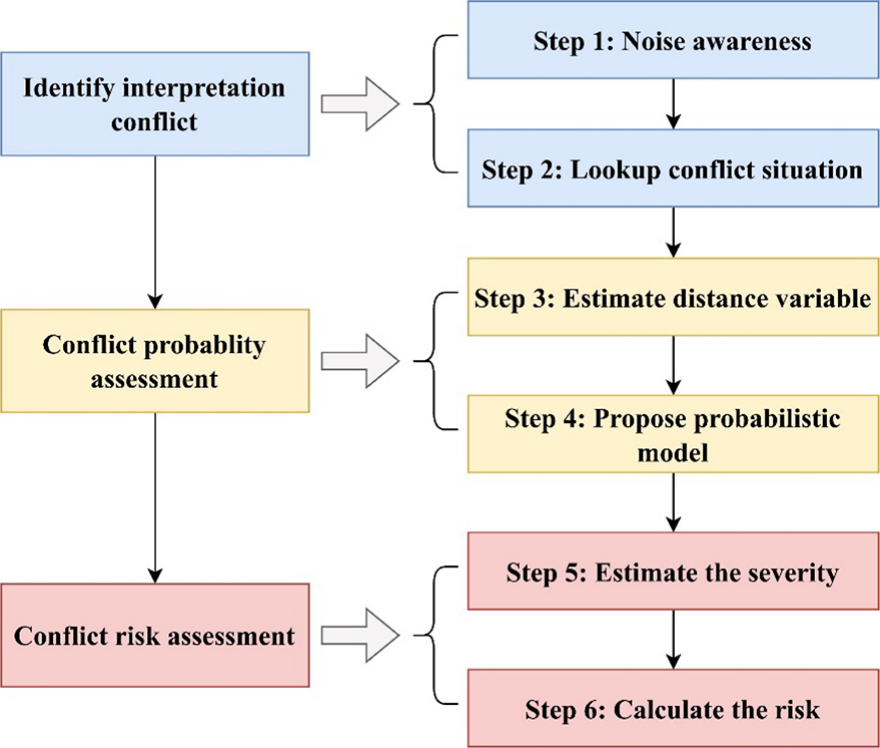
Methodology to assess interpretation conflict risk.

The methodology is shown in Figure 5, and detailed steps are described below.

Step 1: To identify interpretation conflict, first, it is necessary to monitor the process value and be aware of noises, including sensor faults, logic errors, measurement errors, cyberattacks, mistakes, and misunderstandings.

Step 2: In this step, the situations of interpretation conflict are categorized and summarized, and then the lookup method is applied to identify the conflict situations.

Step 3: Based on Bayesian theory and fitted triangular distribution, the interpretation probability is derived. The distance between the vector of AI interpretation probability and the vector of human interpretation probability is measured.

Step 4: The probabilistic model of interpretation conflict is developed in this step.

Step 5: After analyzing the severity distribution, the equation of conflict severity is proposed.

Step 6: The risk is quantified and graded for decision-making.

### 3.2 Identify Interpretation Conflict

#### 3.2.1 Noise Awareness

The interpretation conflict can occur in an instant and is coupled with observation conflict. Usually, the noises could be reflected in the abnormal process values. Hence, the operator is required to monitor any fluctuations and deviations and be aware of sensor faults, logic errors, cyberattacks, measurement errors, mistakes, and misunderstandings. In this study, noise is a broader collective term, which may include white Gaussian noise, random noise, perturbation, disturbance, interference, and error.

#### 3.2.2 Lookup Conflict Situation

**Figure 6:**
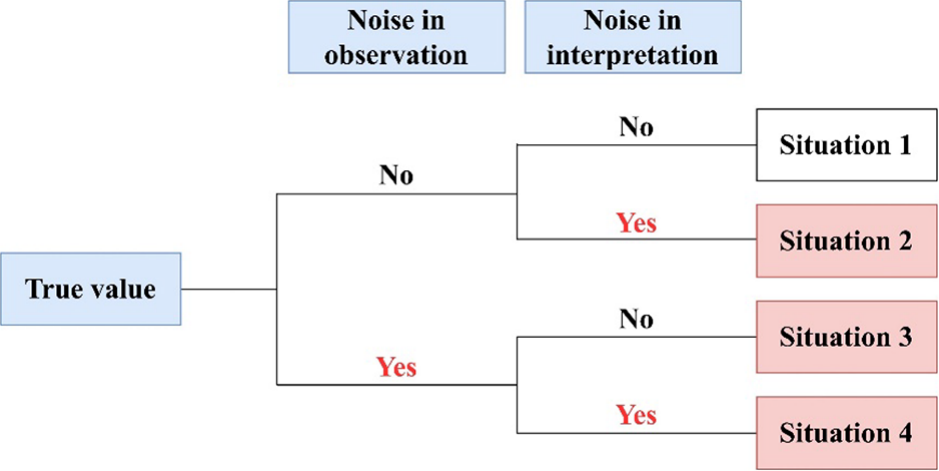
Situations of interpretation conflict.

The lookup method is applied to identify interpretation conflict situations. The classification of conflict situations is shown in Figure 6. In the perfect situation, there is no noise in observation and interpretation; therefore, it is a normal operation without conflicts (Situation 1). Interpretation conflict may arise from noise in interpretation (e.g., logic error or human misunderstanding) (Situation 2). If there is noise in observation, such as measurement error, sensor fault, or human mistake, there may be observation conflict; consequently, it triggers interpretation conflict (Situation 3). When it is small enough (VOD < ± σ), it is acceptable. In some cases, observation noise and interpretation noise may exist together, and the interpretation conflicts overlap (Situation 4). In summary, Situations 2, 3, and 4 are interpretation conflicts.

### 3.3 Conflict Probability Assessment

#### 3.3.1 Estimate the Distance Variable

**Figure 7:**
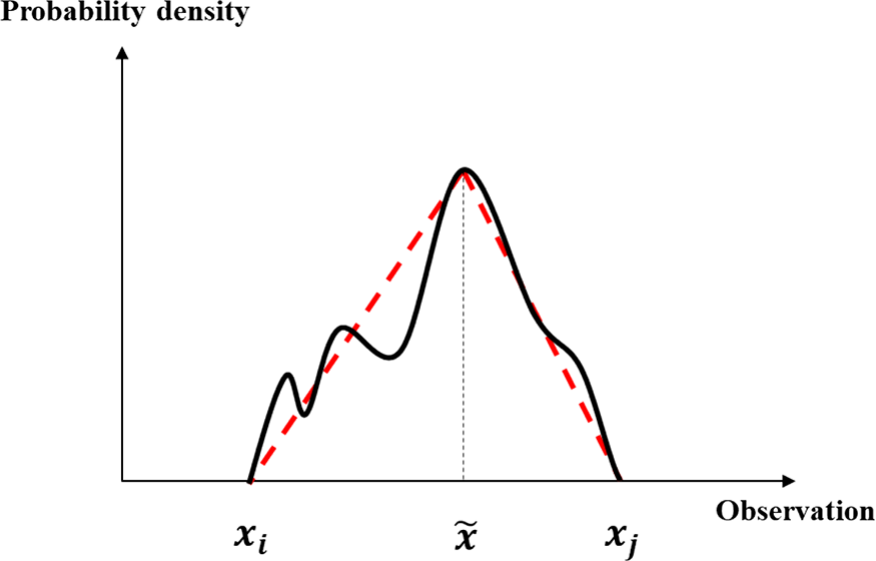
Fitted triangular distribution of observations.

Suppose the observations have a range that a triangular distribution can fit (Figure 7).

Then, the probability of each observation is
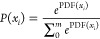
10where PDF is the probability density function of observations.

Similarly, the probability of each interpretation can be estimated as
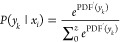
11where PDF^′^ is the probability density function of interpretations, which is another triangular distribution.

The observation determines what the interpretation will be. Therefore, the interpretation result follows the conditional probability rule, and the interpretation probability is
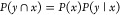
12

For ease of understanding, *P*(*y* ∩ *x*) is simplified as *P*(*y*); for example, *P*(*y_k_*) = *P*(*x_i_*)*P*(*y_k_* | *x_i_*). After transforming to the same size as the action vector, the final vector of AI interpretation probability can be obtained. On the other hand, for humans, it has *P*(*ŷ*) = *P*(*x̂*)*P*(*ŷ* | *x̂*). The probabilities of other interpretation results are marked as 0 to form the vector of human interpretation probability.

Here, it is proposed to measure the distance *d* between the vector of AI interpretation probability and the vector of human interpretation probability.
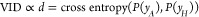
13

Also, VID varies to *d*. The cross entropy is widely applied in deep learning and is more significant compared with other distance algorithms in this study.

**Figure 8:**
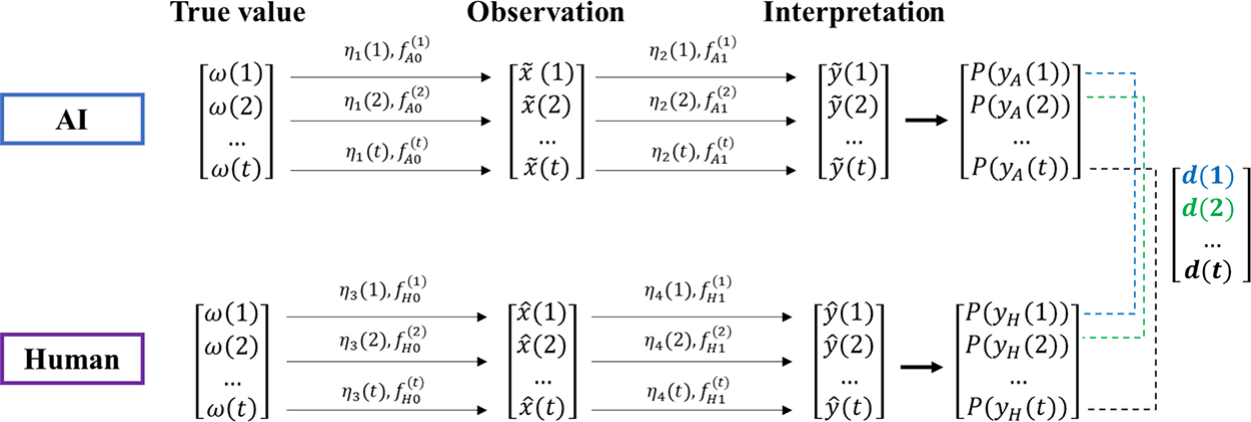
Distance variable of interpretation conflict.

As noise is usually time-varying and the interpretation conflict often lasts for a period, the range of observations may vary from one time step to another. Hence, at each time step, the AI observation function and interpretation function should be different. This statement is also valid for human observation and interpretation. Therefore, for multiple observations in time series, the distance varies with time (Figure 8).

#### 3.3.2 Proposed Probabilistic Model

**Figure 9:**
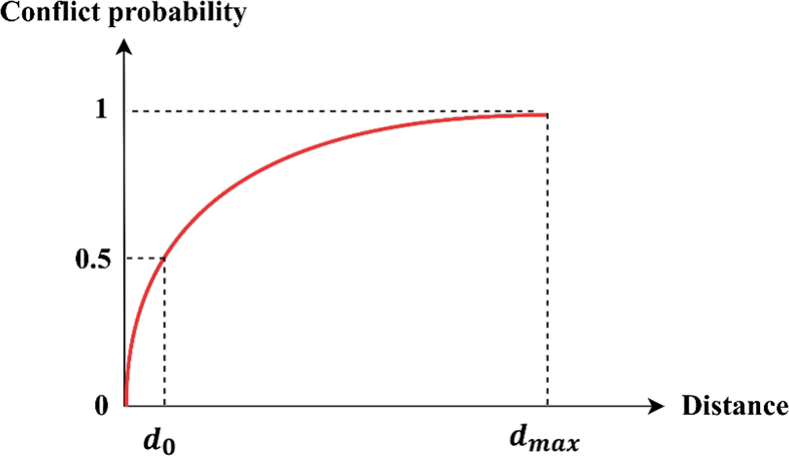
Probability distribution of interpretation conflict.

Based on the above derivation, when *d* = 0, there is no interpretation conflict. There should be a maximum *d*_max_; when *d* = *d*_max_, an interpretation conflict certainly occurs. However, when 0 < *d* < *d*_max_, there is a possibility of an interpretation conflict occurring (Figure 9).

Therefore, the interpretation conflict probability *P* is proposed as
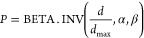
14where α and β are the parameters of the beta inverse distribution. *d*_0_ responds *P* = 0.5; *d*_max_ is associated with the size of the vector, which is the vector distance when the AI and human give different interpretations with 100% confidence, for example,
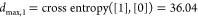
15
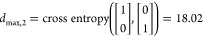
16

Table 1 shows some examples of *d*_max_.

**Table 1:** Example of *d*_max_

vector size	distance
1	36.04
2	18.02
3	12.01
...	...
100	0.36
...	...
1000	0.04

### 3.4 Conflict Risk Assessment

#### 3.4.1 Estimate the Severity

The conflict severity, *S* is proposed as
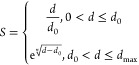
17

**Figure 10:**
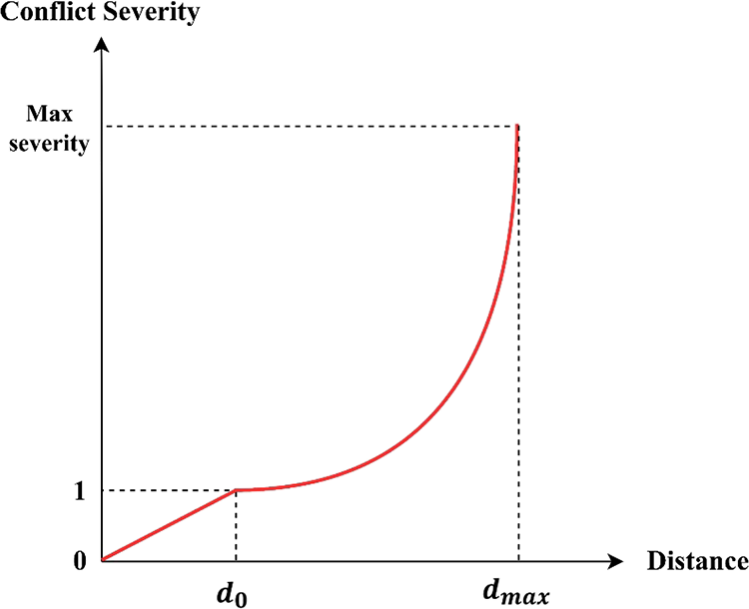
Severity distribution of interpretation conflict.

When 0 < *d* ≤ *d*_0_, the severity follows a linear function; at *d*_0_, the severity is 1; when *d*_0_ < *d* ≤ *d*_max_, the severity follows an exponential function (Figure 10).

#### 3.4.2 Calculate the Risk

Correspondingly, the risk, *R* is
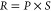
18

The risk can be graded into two categories. When the risk is less than 0.5, the interpretation conflict is acceptable. Otherwise, it is alarming, and action needs to be taken to minimize the risk.

## 4 Application of the Proposed Methodology

### 4.1 Case Description and Simulation

**Figure 11:**
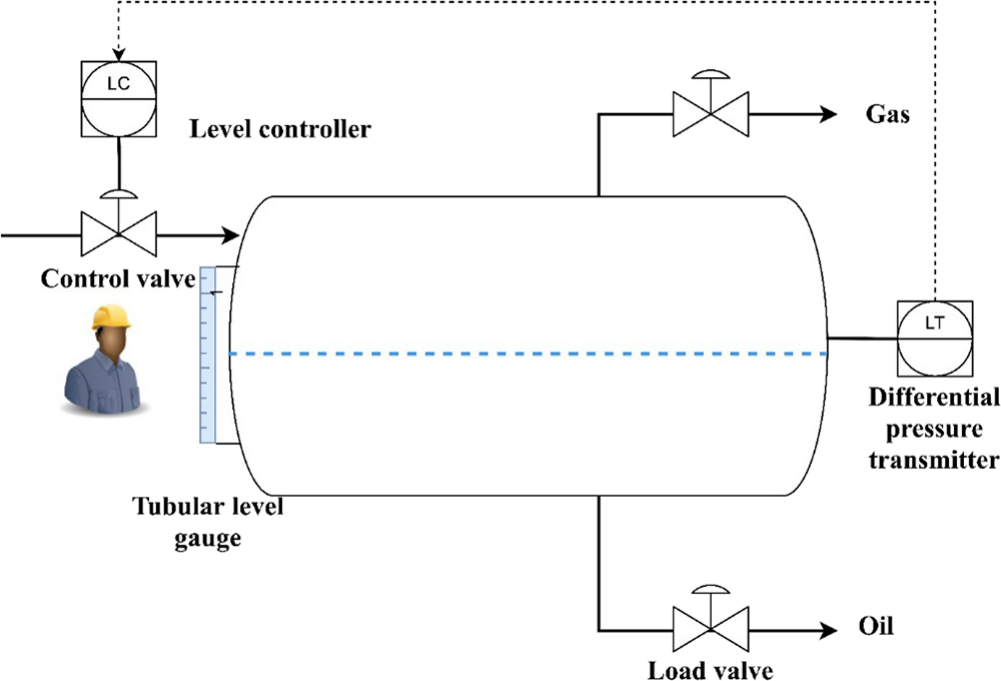
Two-phase oil and gas separator.^28^

The two-phase separator is a common unit to separate oil and gas (Figure 11). This study sets two types of level measurement: a tubular level gauge and a differential pressure transmitter. An operator monitors the system by reading the tubular level gauge. The differential pressure transmitter is connected to the level controller and the control valve.

This study assumes that crude oil has the same density as water. A built-in model in MATLAB is used, and the detailed assumptions can be found in ref (34). The cross-sectional area of the tank, setpoint height of oil in the tank, responding valve opening, the height of the tank, cross-sectional area of the pipe, and maximum inflow rate of oil intake are 1 m^2^, 0.50 m, 50%, 1 m, 0.005 m^2^, and 1 m^3^/s, respectively. The variables and ranges are presented in Table 2. *N* denotes normal distribution.

**Table 2:** Variables of the Two-Phase Separator

variable	symbol	description	range
input	*u*	action: the valve opening	[0,100%]
state	*x*	observation: the height of oil	[0,1]; *x*∼*N*(0.5,0.01^2^)
output	*y*	next time step observation	[0,1]; *y*∼*N*(0.5,0.01^2^)

For the two-phase separator, the differential equation is
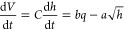
19where *V* is the volume of oil in the tank, *C* is the cross-sectional area of the tank, *h* is the height of oil in the tank, *b* is a constant related to the flow rate into the tank, *q* is the inlet flow rate, and *a* is a constant related to the flow rate out of the tank.

For the subsystem of the inlet valve, it has
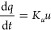
20where *K_u_* is the coefficient constant of the valve opening.

Referring to the built-in model in MATLAB,^34^ the transfer function from the input variable to the output variable in our case is proposed as
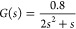
21

**Figure 12:**
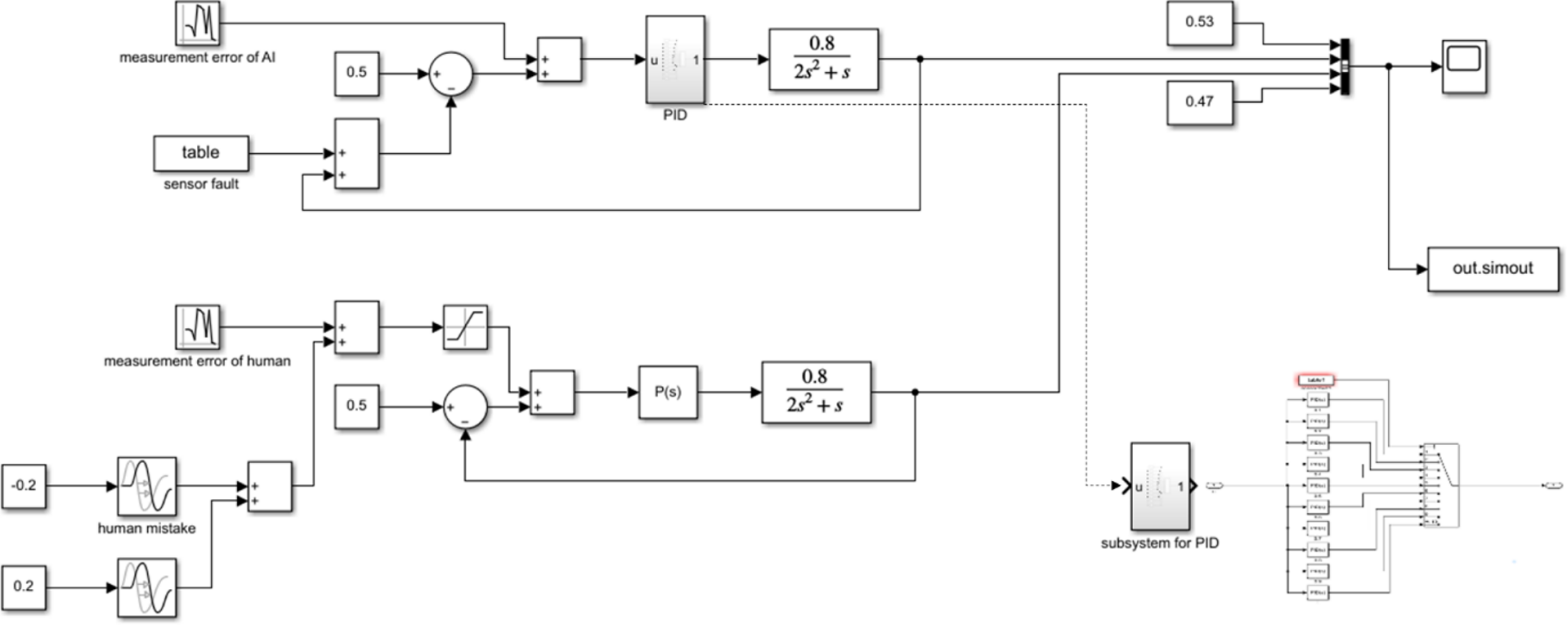
Simulink model of interpretation conflict.

The simulation setup in the MATLAB/Simulink R2021a environment is shown in Figure 12. A proportional-integral-derivative (PID) controller simulates the AI, and a proportional controller simulates the human. The techniques to simulate noises are random number signals representing measurement errors, input table with manipulated observations serving as the sensor fault, addition and subtraction of constant numbers working as human mistakes, and switch modules with different values representing the logic error and human misunderstanding.

### 4.2 Identify Interpretation Conflict

#### 4.2.1 Noise Awareness

As mentioned earlier, the initial task is to find the noise in observation and interpretation. The simulation steps follow the description in Table 3 to add the noise gradually.

**Table 3:** Simulation Steps to Add Noises

time	noise type
1–500 s	No noise
501–1000 s	A sensor measurement error with Gaussian white noise *N*(0,0.001^2^)
1001–1500 s	A human measurement error with Gaussian white noise *N*(0,0.01^2^)
1501–2500 s	A sensor fault to manipulate observations with a triangular distribution [0.2, 0.7, 0.9]; also, an observation mistake by human with −0.2 of each observation at 1701–1710 s
2001–3000 s	A logic error on the PID controller to manipulate proportional value with a triangular distribution [0, 8, 10] (default is 0.2)
2501–3000 s	A misunderstanding on the P controller to change the proportional value to 1 (default is 0.2)

**Figure 13:**
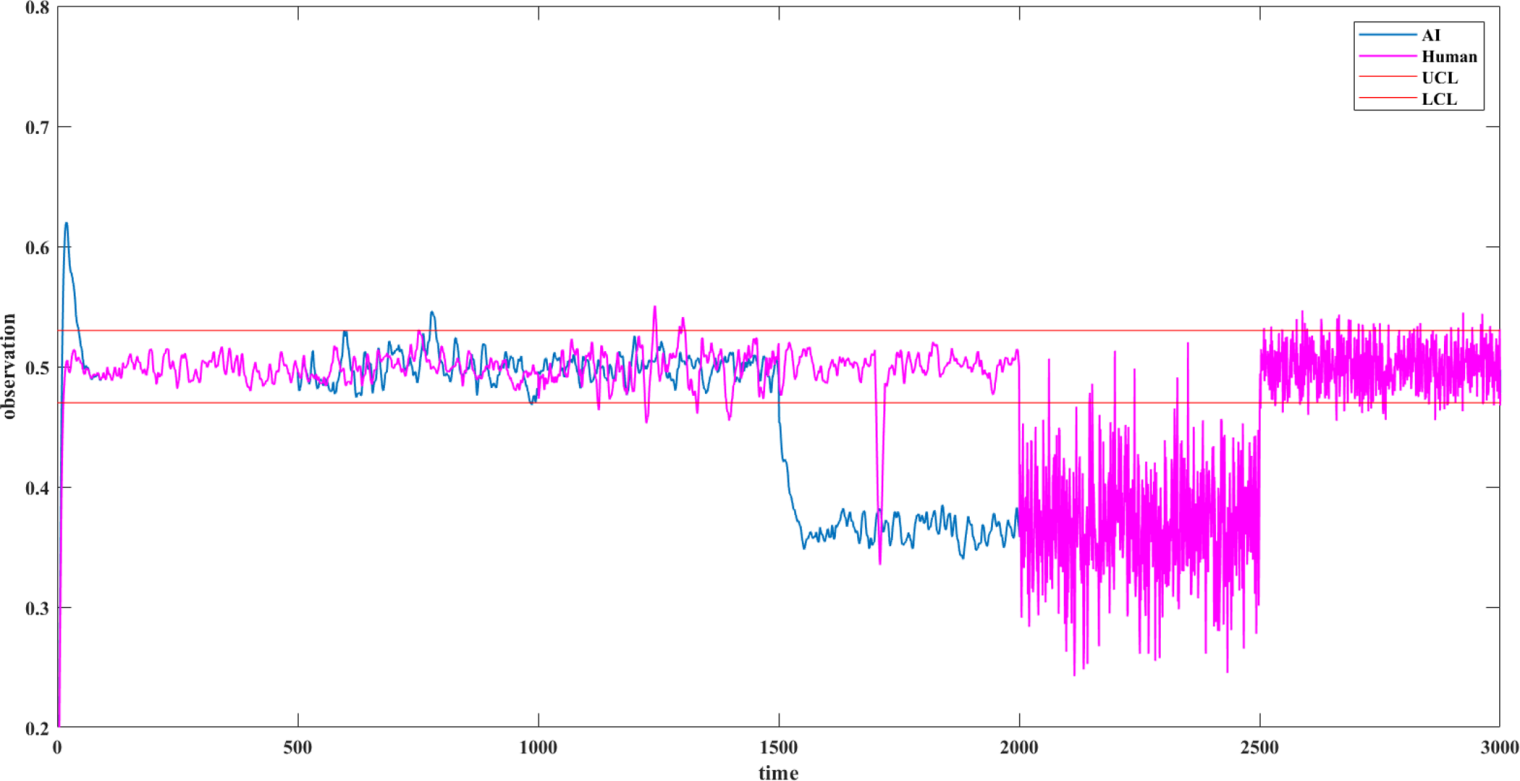
Observations of the oil level.

The simulation results are shown in Figure 13. The sharp variation in the first 80 s is the initial fluctuation to reach a stable state.

**Figure 14:**
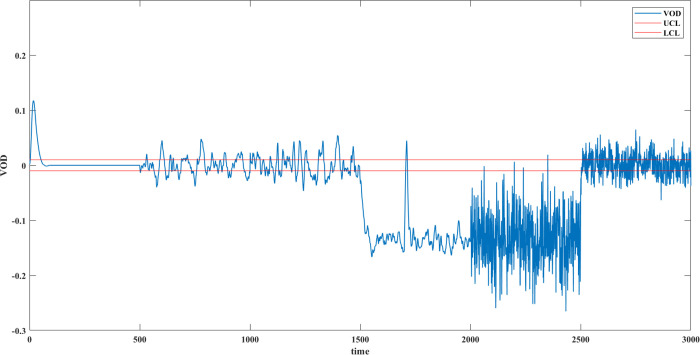
VOD for observation conflict.

Correspondingly, the VOD for observation conflict is obtained (Figure 14).

#### 4.2.2 Lookup Conflict Situation

The situations of interpretation conflict are identified and summarized in Table 4.

**Table 4:** Identification Results of Interpretation Conflict

time	conflict situation	interpretation conflict
1–500 s	Situation 1	no
501–2000 s	Situation 3	yes
2001–2500 s	Situation 4	yes
2501–3000 s	Situation 2	yes

Situation 1: In 0–500 s, as there is no noise at any time, human observation and sensor observation are the same. There is no interpretation conflict.

Situation 3: In 501–1000 s, as there is a sensor measurement error, the sensor observations deviate from the true values. However, they are still mostly between control limits. In 1001–1500 s, the human measurement error makes observations deviate from the reference values. In both periods, observation conflict persists for a few instances that may trigger interpretation conflict. This is a situation that is described as confusion.

In 1501–2000 s, a sensor fault with a triangular distribution [0.2, 0.7, 0.9] is added. As most observations are higher than the setpoint 0.5, the controller takes action to adjust. It results in a low liquid level. It causes observation and interpretation conflicts. This is an automation surprise.

In 1701–1710 s, an observation error happens from the operator end, which makes the observation curve sharply deviate from the true value. In most cases, such a mistake stays for a short period, and the operator may become aware of it later. Observation and interpretation conflicts also occur in such situations.

Situation 4: In 2001–2500 s, a logic error on the PID controller to manipulate proportional value with a triangular distribution [0, 8, 10] (default is 0.2) occurs. It makes the controller lose its accuracy in adjusting the liquid level. Together with the sensor fault, observation conflict and overlapped interpretation conflict occur.

Situation 2: In 2501–3000 s, the operator finds the cause of the sensor fault and solves it. However, the fluctuation keeps occurring because the logic error is still present. The operator misunderstands the situation and takes a wrong action, which is simulated by changing the proportional value of the proportional controller to 1 (default is 0.2). Human observations fluctuate beyond the limit. An interpretation conflict occurs.

### 4.3 Conflict Probability Assessment

#### 4.3.1 Calculate the Distance in 2001 s

As the logic error occurs in 2001–3000 s, we select the time step 2001 s as the research object, where sensor observation is 0.36, and the proportional value of PID is 6. In this simulation, as the switch modules are used to represent the shift between logic decisions (proportional value of PID), therefore, once determined, the proportional value is certain, with a certain probability, and other probabilities are 0. According to eqs 10–13, each variable and value is calculated and shown in Table 5.

**Table 5:** Calculation Results at 2001 s

variable	value
*P*(*x*)	6.2 × 10^–03^
*P*(*y* | *x*)	1.0 × 10^–02^
*P*(*y*)	6.4 × 10^–05^
*P*(*y_A_*(2001))	[0,0, ...,6.4 × 10^–05^, ...,0]^*T*^
*P*(*y_H_*(2001))	[0,0, ...,1.4 × 10^–04^, ...,0]^*T*^
*d*_max_	3.6 × 10^–01^
*d*	2.3 × 10^–05^

#### 4.3.2 Calculate the Probability in 2001 s

According to eq 14, suppose α is 20 and β is 1, then the conflict probability at time 2001 is
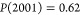


### 4.4 Conflict Risk Assessment

According to eqs 17 and 18, for the time step 2001 s, the severity and risk are
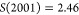

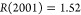


**Figure 15:**
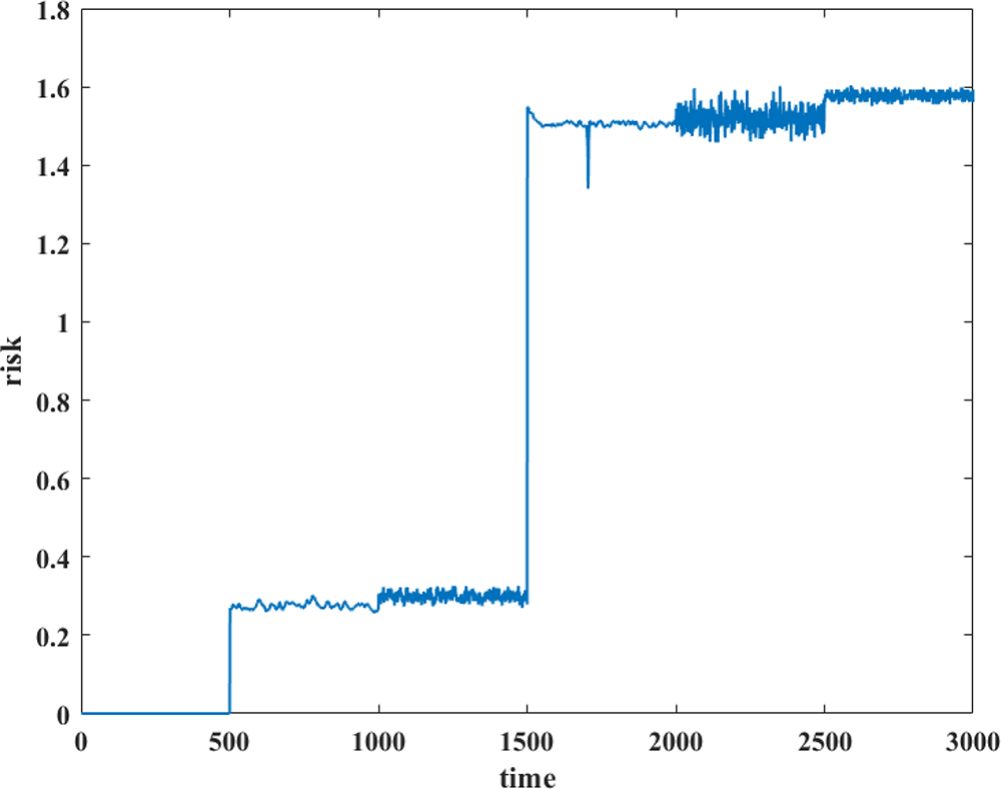
Risk in 0–3000 s.

The risk is much greater than 0.5, and it can be concluded that interpretation conflict occurred in 2001 s, just at the same time when the logic error happened. In addition, the risk of the whole period is calculated and shown in Figure 15.

In 501–1000 s (sensor measurement error) and 1001–1500 s (human measurement error), the risk is less than 0.5, which can be considered relatively small, and the risk of interpretation conflict is acceptable. In 1501–2000 s, the sensor fault increases the risk sharply. When it overlaps with logic error in 2001–2500 s, the risk increases even higher. In 2501–3000 s, the logic error overlaps with the human misunderstanding, making the risk fluctuate further.

Such a real-time risk figure displays how interpretation conflict behaves in different situations. When the interpretation conflict risk appears high, it is time to consider whether an interpretation conflict has occurred rather than always a fault or failure. Operators are thus better able to take more targeted measures to resolve the conflict.^28^ Typically, the violent fluctuations are more likely to be the superposition of observation conflict and interpretation conflict, such as Situation 4 in 2001–2500 s, whereas a lower risk may indicate common measurement errors, e.g., Situation 1 in 501–1500 s.

## 5 Discussion

From the above sections, the following key points can be emphasized and discussed.

### 5.1 Noise Effect on Observation and Observation Conflict

Severe observation conflict may occur when the observations deviate from the setpoint significantly. Additionally, the VOD is clearly beyond the limit once a noise is introduced, including measurement error, sensor fault, logic error, and human mistake and misunderstanding. This is common in process operations, and it implies that real-time monitoring and response are essential.

### 5.2 Difficulty to Identify and Assess Interpretation Conflict

From the human response perspective, interpretation conflict is expressed as confusion, like mode confusion and automation surprise. As the operators can only judge and interpret from observations, the observations cannot indicate the interpretation conflict alone. This confirms that the logic errors and cyberattacks on the logic solver or AI model are usually hidden and invisible. On the other hand, from the risk assessment results of the time step 2001 s, an interpretation conflict is instant once the logic error happens, and the risk reaches high sharply. Therefore, it is necessary to use risk-based approaches to predict and assess it.

### 5.3 Noise Effect on Interpretation Conflict

In 2001–3000 s, the logic error happens. Once the interpretation conflict occurs, it is easy for the operator to misunderstand the situation and take the wrong action. Such noise may have different types and forms; traditionally, it may be the mechanical problem or programming problem of the logic solver. Any other interference or impairment of the computing capability, for example, DoS attacks, might have a similar interpretation conflict. Usually, data pollution, insufficient data volume, and limited training can degrade AI’s applicability, integrity, and robustness. Consequently, they may force the AI to interpret incorrectly, which needs further verification.

### 5.4 Bounded Noise or Unbounded Noise

The traditional control theory solves the disturbance of bounded noise well. However, the noises caused by sensor faults, cyberattacks, and human errors are usually unbounded, especially, from the security perspective. These noises may have similar fluctuations in the observations. In this study, the triangular distribution is proposed to set the boundaries of the noise. From the time series, the observations are quite fluctuating and hide the interpretation conflict. This can be challenging for inexperienced operators to judge. This also confirms that there are some undetectable logic problems, or the hacker is reluctant to let the operator find obvious abnormalities.

### 5.5 Distance to Measure Interpretation Conflict

Measuring the distance of probability vectors between humans and AI to measure the interpretation conflict is the most challenging part of this study since interpretation is intangible. This refers to the techniques in deep learning, which usually use Softmax to obtain the probability vector and cross-entropy to measure the loss. Compared to the Manhattan and Euclidean distances, cross entropy-based distance measurement is found suitable for interpretation conflict assessment.

### 5.6 Resistance of Advanced Control and Data-Driven Control to Interpretation Conflict

This study employs s a linear model-based control (i.e., PID) on a classic model with a single input and output to show how various noises generate interpretation conflicts. The reason for choosing PID instead of more advanced or even data-driven control is that PID is still the primary choice in process industries. One hypothesis is that advanced control (e.g., model predictive control) or AI control might counteract or respond differently to interpretation conflicts. Especially for the time series data, recurrent neural networks (RNN) and their variants can be suitable to buffer the disturbances. In the meantime, performance indicators of AI models can evaluate the noise effect and may contribute to estimating the conflict, which needs further study. Eventually, if the noise/disturbance can be suppressed, it may not trigger human–AI conflict. On the other hand, AI algorithms usually display the black-box issue; therefore, combining physical model-based control and data-based control may produce better performance, yet challenging.

## 6 Conclusions

This study deconstructs the cognitive processes of humans and AI by proposing the concept of interpretation conflict, extending the situation awareness to interpretation conflict, and proposing the methodology to identify the situations of interpretation conflict, further evaluating its probability and quantifying its severity and risk. The proposed methodology has been applied to a two-phase separator unit. The simulation shows that when interpretation conflict occurs, the observations are quite similar to traditional faults. Significant observation conflict triggers interpretation conflict. Also, various noises can cause interpretation conflict, including sensor faults, logic errors, cyberattacks, human mistakes, and misunderstandings. When there is an interpretation conflict, humans may not take the right action timely, allowing a conflict to lead to catastrophic consequences.

This paper emphasizes the need for assessing interpretation conflict to discover the difference between intelligence control and human-centric control to optimize the controller design from a safety perspective. Considering interpretation conflict as unbounded noise provides a broader idea for model predictive control and other data-driven control design. As intelligent machines approach full automation, situation awareness becomes critical. Incorporating this in design and operation will help achieve safer and more robust processes. This study does not consider multiple inputs and multiple outputs. This is an essential aspect of AI and how humans will consider multiparameter data (sensor data fusion) differently. This is a future research direction.

## NOMENCLATURE

*a*: a constant related to the flow rate out of the tank; subscript of interpretation
α: parameter of a Beta distribution
*b*: a constant related to the flow rate into the tank; subscript of interpretation
β: parameter of a Beta distribution
BETA. INV: return the inverse of the beta cumulative probability density function
*C*: cross-sectional area of the tank
*d*: distance between the vector of AI interpretation probability and the vector of human interpretation probability
*f*: function from observation to action
*f*_0_: function from true value to observation
*f*_*A*0_: function from true value to sensor observation
*f*_*H*0_: function from true value to human observation
*f*_1_: function from observation to interpretation
*f*_*A*1_: function from observation to interpretation of AI
*f*_*H*1_: function from observation to interpretation of human
*f*_2_: function from interpretation to action
*G*: transfer function from input to output
*h*: height of oil in the tank
*K_u_*: coefficient constant of the valve opening
*m*: observation vector size
*n*: action vector size; the transformed interpretation vector size
*N*: normal distribution
η: noise
η_1_: noise in sensor observation
η_2_: noise in AI interpretation
η_3_: noise in human observation
η_4_: noise in human interpretation
ω: true value
*P*: conflict probability
*q*: inlet flow rate
*R*: conflict risk
*s*: complex variable
*S*: conflict severity
*t*: time
*u*: action or input; valve opening
*ũ*: most possible AI action
*û*: most possible human action
*V*: volume of oil in the tank
*x*: observation
*x̃*: most possible sensor observation
*x̂*: most possible human observation
*y*: interpretation; next time step observation
*ỹ*: most possible AI interpretation
*ŷ*: most possible human interpretation
